# Exploring pineapple peel hydrolysate as a sustainable carbon source for xylitol production

**DOI:** 10.1038/s41598-023-46061-8

**Published:** 2023-11-07

**Authors:** Nur Zahidah Nasoha, Abdullah Amru Indera Luthfi, Muhammad Faizuddin Roslan, Hikmah Bajunaid Hariz, Nurul Adela Bukhari, Shareena Fairuz Abdul Manaf

**Affiliations:** 1 Department of Chemical and Process Engineering, Faculty of Engineering and Built Environment, Universiti Kebangsaan Malaysia (UKM), 43600 Bangi, Selangor Malaysia; 2 Research Centre for Sustainable Process Technology (CESPRO), Faculty of Engineering and Built Environment, Universiti Kebangsaan Malaysia, 43600 Bangi, Selangor Malaysia; 3 Energy and Environment Unit, Engineering & Processing Research Division, Malaysian Palm Oil Board (MPOB), 6, Persiaran Institusi, 43000 Bandar Baru Bangi, Kajang, Selangor Malaysia; 4 School of Chemical Engineering, College of Engineering, Universiti Teknologi MARA, 40450 Shah Alam, Selangor Malaysia

**Keywords:** Fungi, Cell growth

## Abstract

This study explores utilizing pineapple peel (PP) hydrolysate as a promising carbon source for xylitol production, covering scopes from the pre-treatment to the fermentation process. The highest xylose concentration achieved was around 20 g/L via mild acid hydrolysis (5% nitric acid, 105 °C, 20-min residence time) with a solid loading of 10%. Two sets fermentability experiments were carried out of varying pH levels in synthetic media that includes acetic acid as the main inhibitors and hydrolysate supplemented with diverse nitrogen source. The results revealed that pH 7 exhibited the highest xylitol production, yielding 0.35 g/g. Furthermore, urea was found to be a highly promising and cost-effective substitute for yeast extract, as it yielded a comparable xylitol production of 0.31 g/g with marginal difference of only 0.01 g/g compared to yeast extract further highlights the viability of urea as the preferred option for reducing xylitol production cost. The absence of a significant difference between the synthetic media and hydrolysate, with only a marginal variance of 0.35 to 0.32 g/g, implies that acetic acid is indeed the primary constraint in xylitol production using PP hydrolysate. The study sheds light on PP biomass's potential for xylitol production, aligning economic benefits with environmental sustainability and waste management.

## Introduction

Malaysia stands as a significant agricultural producer in Southeast Asia, yielding around 335 × 10^3^ tons of pineapples annually which contributes to by-products of 40% for the peel and 20% for the leaf^1^. The great amount of discarded pineapple peel (PP) contributes to the greenhouse gas effect when discarded in landfills. Addressing this, many researchers have worked together to find the potential of these by-products for conversion into a high-value product. It has been discovered that these byproducts contain valuable components like bromelain, which breaks down proteins for various uses^2,3^, polyphenols with health benefits^4^, and sugars that can fuel microbes to produce valuable products like organic acids and alcohol^1^. Moreover, beyond its conventional role as agricultural residue, PP harbors untapped potential to revolutionize various sectors, from bioenergy production^5^ to eco-friendly surfactant manufacturing^6^. However, in the realm of fermentation processes, it is not yet scrutinized its application on xylitol production. Xylitol finds applications across food, confectionery, cosmetics, pharmaceuticals, and other sectors. Notably, Malaysia accounts for 40% of global xylitol output, with demand projected to reach 50% of the total market value, amounting to US$1.1 × 10^9^ by 2025^7^.

While current xylitol production relies on costly chemical approaches like catalytic hydrogenation, biotechnological xylitol production is considered more efficient and cost-effective due to its lower energy consumption^8^. To offer a greener and more sustainable alternative in xylitol production, the extraction of xylose, a key component, from lignocellulosic biomass residues has gained attention. This provides an eco-friendly substitute for synthetic sources. Various lignocellulosic biomasses, such as soybean hull^9^, sugarcane bagasse^10^, and corn stover^11^, have been explored for xylose recovery. Interestingly, PP, containing 22–45% dry matter cellulose, 21–75% dry matter hemicellulose, and 2–14% dry matter lignin^12^, falls within the same range as other biomass sources used for xylitol production. This similarity positions PP as a strong candidate for sustainable fermentable sugar production, specifically xylose.

The heart of xylitol production extends beyond mainstream fermentation, encompassing upstream processing to secure a sustainable and efficient xylose carbon feedstock. In this process, the rigid biomass structure must be broken down to access the sugar inside it. A spectrum of pre-treatment methods, encompassing chemical and enzymatic approaches, have been employed to extract xylose sugar from the biomass^13^. Among these methods, acid hydrolysis has emerged as a highly effective and cost-efficient technique for xylose sugar extraction. Mild acid hydrolysis, in particular, has gained attention due to its ability to efficiently extract xylose while reducing inhibitor concentrations^14,15^. This quick and concurrent hydrolysis, while decreasing inhibitor concentration, relies on the variation of three key parameters: acid concentration, temperature, and residence time, all influenced by biomass composition^16^. The variability of these parameters underscores the need to customize the processing approach for optimal results.

Considering the importance of upstream processing and the role of dilute acid treatment in boosting xylose recovery, addressing the consequences of this pretreatment method is crucial. In particular, the formation of inhibitors like furfural, 5-hydroxymethylfurfural (5-HMF), and acetic acid must be acknowledged, as they can hinder cell growth and impact xylitol production. Due to that reason, the previous study that utilised performed detoxification process before the fermentation process. For instance, Jin et al.^17^ conducted a study using detoxified hydrolysate of Quinoa straw and achieved a substantial 19% increase in xylitol yield compared to non-detoxified samples (0.42 g/g to 0.50 g/g). This example clearly highlights how the removal of inhibitors plays a crucial role in optimizing xylitol production. Additionally, Cheng et al.^18^ illustrated the impact of high acetic acid concentration (4 g/L), resulting in a 72% lower xylitol production (0.72 g/g to 0.5 g/g). These examples underscore the significance of inhibitor removal in enhancing xylitol production. While inhibitor removal is discussed in various studies, some researchers highlight the challenges of detoxification due to cost and time constraints^19^.

In order to combat the issue, pH adjustment during fermentation emerges as a practical alternative, countering inhibitors’ effects. For instance, previous study revealed that raising the pH from 4.5 to 6 reduces the undissociated form of acetic acid in hydrolysate, lessening its inhibitory impact^18^. In fact, this adjustment resulted in a twofold increase in xylitol concentration. However in the previous study, it only study the effect of the pH adjustment on the lowest tolerance of the acetic acid concentration. As of our best knowledge, there is no existing study that has investigated the impact of pH adjustment beyond the threshold of the microbes in the context of xylitol production especially when acetic is the commonly exist is acid hydrolysis pre-treatment.

Nitrogen source selection is another pivotal factor in xylitol fermentation. Inhibitors present in the hydrolysate not only impede microbial growth but also disrupt yeast metabolic processes and influence gene expression related to nutrient uptake^20^. This nitrogen source catalytically affects enzymes involved in xylose metabolism, like xylose reductase and xylitol dehydrogenase, with variations based on biomass and microbe characteristics^21^. Amid these considerations, optimizing fermentation parameters, specifically pH and nitrogen source, becomes critical. The goal is to identify pH and nitrogen sources that synergize effectively with PP hydrolysate, leading to enhanced xylitol production by addressing inhibitor challenges and maximizing fermentation efficiency.

Given this backdrop, the research objective is to evaluate hemicellulose and cellulose degradation in PP for highly fermentable sugar production. Optimizing hydrolysis conditions, including key parameters’ variations, aims to achieve high xylose recovery from PP. Subsequently, PP’s fermentability is tested with *Candida tropicalis*, a commonly used yeast in xylitol production due to its natural possession of xylose reductase (XR) for efficient xylose uptake and xylitol production^8^. Assessing xylose-to-xylitol conversion through fermentation with *C. tropicalis*, even with inhibitors present, determines PP’s suitability for xylitol production. This study’s significance lies in offering preliminary insights into PP’s potential as a xylitol production carbon feedstock and assessing its viability, despite its antioxidant-rich nature^22^.

## Results and discussion

### Composition of pineapple peel (PP)

The PP was characterized using the NREL technique. The PP average composition consisted of 42.9% cellulose, 20.7% hemicelluloses, 9.4% lignin, 2.7% ash and 18.8% total extractives, as shown in Table 1. Compositions from various sources were also presented and compared. In comparison to previous studies, the result has the most parallels to this study^17^. This variation of the result was due to several factors such as variant source of pineapples, place of origin, growing seasons, method of collections, storage temperatures and maturity of the pineapples^18^.

**Table 1:** Overview of PP characterization of other sources.

Cellulose (%)	Hemicellulose (%)	Total structural carbohydrates (%)	Lignin (%)	Ash (%)	Crude protein (%)	Total extractives (%)	References
42.9	20.7	63.6	9.4	2.7	4.6	18.8	This study
40.55	28.69	69.24	10.01	1.5	0.75	–	^17^
20.9	31.8	52.7	10.4	5.9	3.9	28.1	^19^
32.44	23.2	55.64	19.4	5.27	–	14.5	^20^
35	19.7	54.7	16	4.7	0.33	–	^21^

### Nitric acid hydrolysis optimization

**Figure 1:**
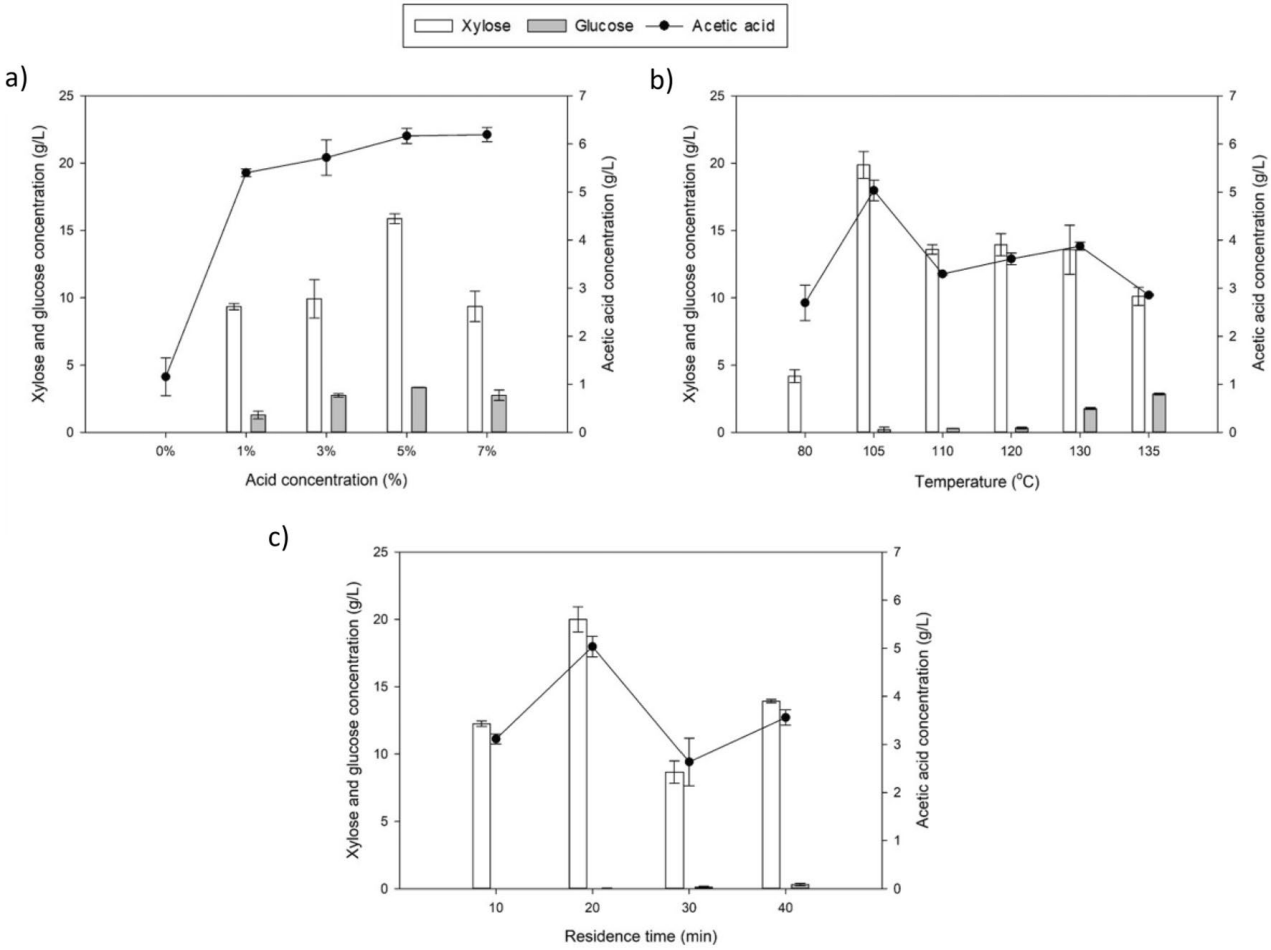
The effect of (**a**) concentration, (**b**) temperature, and (**c**) residence time on the production of xylose, glucose and inhibitor, acetic acid. The values plotted were expressed from three independent experiments expressed as mean ± standard deviation below than 5 g/L.

Figure 1 illustrated the recovery of xylose, glucose and acetic acid formation from three parameters varied; concentration of acid, temperature and residence time. It was determined that the maximum xylose sugar concentration could be produced by using 5% acid concentration at 105 °C for 20 min residence time that produced approximately up to 20 g/L xylose which resulted in to 85% recovery of the xylose from the actual potential xylose sugar in the hemicellulosic. The graphs presented in Fig. 1a–c illustrate the effects of the acid concentration, temperature, and residence time as independent variables respectively toward the xylose and other byproducts. Interestingly, in this study the main inhibitors that mostly been reported in acid hydrolysis (furfural and 5-HMF) were present in very low concentrations, thus their presence in the graph was not shown. The analysis revealed that 5-HMF was undetectable, while furfural was detected at a concentration of 0.05 g/L under the optimized parameters (5% acid concentration, 105 °C temperature, and 20 min residence time). Thus, this study suggests that the acid hydrolysis conditions employed were optimized, by the minimum formation of furfural and 5-HMF. This finding of this PP hydrolysate is very interesting as the inhibitors are one of the issues that cannot be neglected during fermentation as the presence of these inhibitors inhibit the growth of microorganisms^22^. The lower concentration of furan derivatives furfural and HMF in this hydrolysate is a very favorable condition in the fermentation process, as it requires no additional pre-treatment for detoxification.

#### Effect of acid concentration

For the first stage of OFAT hydrolysis, which focused on varying the concentration of acid at 130 °C for 20 min, the results are shown in Fig. 1a. The highest sugar concentration of xylose obtained was 15.88 ± 0.37 g/L at a 5% acid concentration. As the acid concentration increased up to 7%, the xylose concentration appeared to decrease, while the concentration of acetic acid increased in proportion to the nitric acid concentration.

The observation also revealed the effectiveness of mild acid hydrolysis in degrading hemicellulose, with a 40% degradation at the lowest acid concentration, increasing up to 67% at 5% nitric acid. A further increase to a 7% acid concentration resulted in a shorter sugar decomposition reaction time, contributing to the final result of reduced sugar yield. This pre-treatment illustrates how lucky the PP hydrolysate is, as it exhibited insignificant glucose production despite cellulose constituting a higher percentage than glucose.

This result further highlights the effectiveness of mild acid hydrolysis in degrading hemicellulose, which predominantly yields xylose due to the weaker ester bonds in hemicellulose compared to the hydrogen bonds in cellulose^23^. This aligns with previous studies that have indicated mild acid hydrolysis resulted in higher xylose concentration compared to glucose, despite cellulose being the predominant constituent in materials like corncobs^24^ and oil palm fronds^15^.

#### Effect of temperature

For the next stage of OFAT hydrolysis, the focus shifted to determining the optimum temperature for a 5% nitric acid concentration over a duration of 20 min. As depicted in Fig. 1b, the concentration of xylose showed an increasing trend with the rise in temperature. However, it exhibited a decline upon reaching 110 °C. Interestingly, the pattern of xylose and acetic acid concentrations followed a similar trend, characterized by fluctuations in their levels with temperature changes.

In contrast, glucose exhibited a consistent increase with temperature elevation, showing a significant rise at 130 °C (0.2 g/L to 2.8 g/L). However, even at 105 °C, the glucose concentration remained relatively low, which is preferable for xylitol production, reaching only 0.2 g/L This similarity between xylose and acetic acid can be attributed to their common origin from the biomass constituent known as hemicellulose.

As the temperature increased while maintaining the same duration, the rate of the decomposition reaction also accelerated. Consequently, the reaction time decreased, leading to a rapid degradation of sugars, and this explains the sudden drop in xylose concentration observed after 105 °C, representing around a 40% reduction. The results obtained from our observation are consistent with previous study which involved hydrolysis using dilute phosphoric acid on potato peel^25^. In that study, a 60% reduction was reported when comparing the optimal temperature to the highest temperature studied (135 °C).

Furthermore, the aforementioned study also demonstrated that the rate of the decomposition reaction increased with higher temperatures. While the reaction time decreased, it resulted in a rapid rate of sugar degradation, eventually surpassing the sugar production rate at elevated temperatures. It is evident that an increase in temperature can significantly affect the rate of sugar production, suggesting that high temperatures may have a detrimental impact on overall sugar yield.

#### Effect of residence time

In the final stage of OFAT hydrolysis, we varied the residence time at a 5% acid concentration and 105 °C. The results depicted in Fig. 1c demonstrated an optimum xylose concentration of 20 g/L achieved at 20 min of residence time. However, as the residence time increased to 30 min, the xylose concentration decreased to 8.6 g/L, representing a 55% reduction.

The pattern of xylose and acetic acid exhibited a similar fluctuating trend as observed in the previous stage when temperature was varied, confirming their interrelated production during the hydrolysis process. On the other hand, the concentration of glucose did not exhibit significant changes with varying residence times but instead showed a steady increase. At the longest residence time of 40 min, the glucose concentration reached 0.38 g/L, while at the optimized residence time of 20 min, it was below 0.1 g/L. This indicates that residence time has a relatively lower impact on cellulose degradation compared to acid concentration and temperature. In contrast, glucose exhibited a consistent increase with temperature elevation, showing a significant rise at 130 °C, reaching 2.8 g/L.

Based on these results, it is revealed that the hemicellulosic fraction depolymerizes faster at lower temperatures than the cellulose fraction with dilute acid treatment. However, at higher temperatures or longer retention times, the formed monosaccharides further hydrolyze into other compounds^26^. Consequently, a residence time of 20 min is identified as the optimal duration for xylose recovery in future applications.

### Performance of xylitol fermentation of varied pH in highly acetic acid hydrolysate

The experimental results presented in Table 2 provide an overview of the influence of varied pH parameters ranging from 4 to 7 on xylitol and biomass yield in high acetic acid concentration. As the pH increases, there is a clear trend of increasing xylitol yield of 30%, peaking at 0.35 g/g at pH 7.0. However, the trend for biomass yield is not consistent, with the highest yield observed at pH 5.0 (0.55 g/g) with ~ 60–70% increment from its previous and later pH. This result illustrates how much the metabolic pathway of xylose utilization is more toward biomass favor at certain pH, while xylitol yield in this study is favored by the increasing of the pH.

**Table 2:** Result of xylose bioreduction to xylitol and microbial growth of PP synthetic media by C. tropicalis at different pH values.

pH	Time (h)	Xylitol concentration, (g/L)	Xylose consumption (g/L)	Dry cell weight (g/L)	Xylitol yield, (g/g)	Biomass yield, (g/g)
4*	96	4.88	17.74	6.73	0.27	0.38
4.5	96	4.25	17.84	5.73	0.24	0.32
5	96	5.07	19.25	10.53	0.26	0.55
5.5	96	5.10	19.63	6.87	0.26	0.35
6	96	5.06	18.69	6.20	0.27	0.33
6.5	96	6.18	19.29	6.60	0.32	0.34
7	96	6.63	19.19	6.73	0.35	0.35
7**	96	9.40	19.87	7.20	0.47	0.36

*Initial pH without any pH adjustment.

**Control without any present of acetic acid.

It merits attention that the present study signifies the preeminence of pH 5.0 in engendering the highest biomass yield, with an ensuing decrement in biomass yield upon transgressing the pH threshold of 5.0. It is worth noting that numerous studies have indicated that yeast strains normally thrive in mildly acidic conditions due to the presence of H+ ions, which favor the XR reaction by increasing the NADH to NAD+ ratio^27^. This is supported by a study that optimised pH for different yeast strains using xylose as their only carbon source. *C. parapsilosis* grew optimally at pH 5.0, *C. guilliermondii* at pH 4.5, *C. boidinii* at pH 6.0, and *H. anomala* at pH 4.5, demonstrating a preference for acidic environments^28^. However, contextualizing the current research, it is pertinent to acknowledge that the optimal pH for enhanced xylitol production in the presence of inhibitors such as acetic acid deviates from the acidic pH ambit. The rationale behind this anomaly lies in the observation that elevating pH levels curtails the undissociated fraction of acetic acid, thereby attenuating its inhibitory effects and in effect, augmenting xylitol biosynthesis^24,29^.

**Figure 2:**
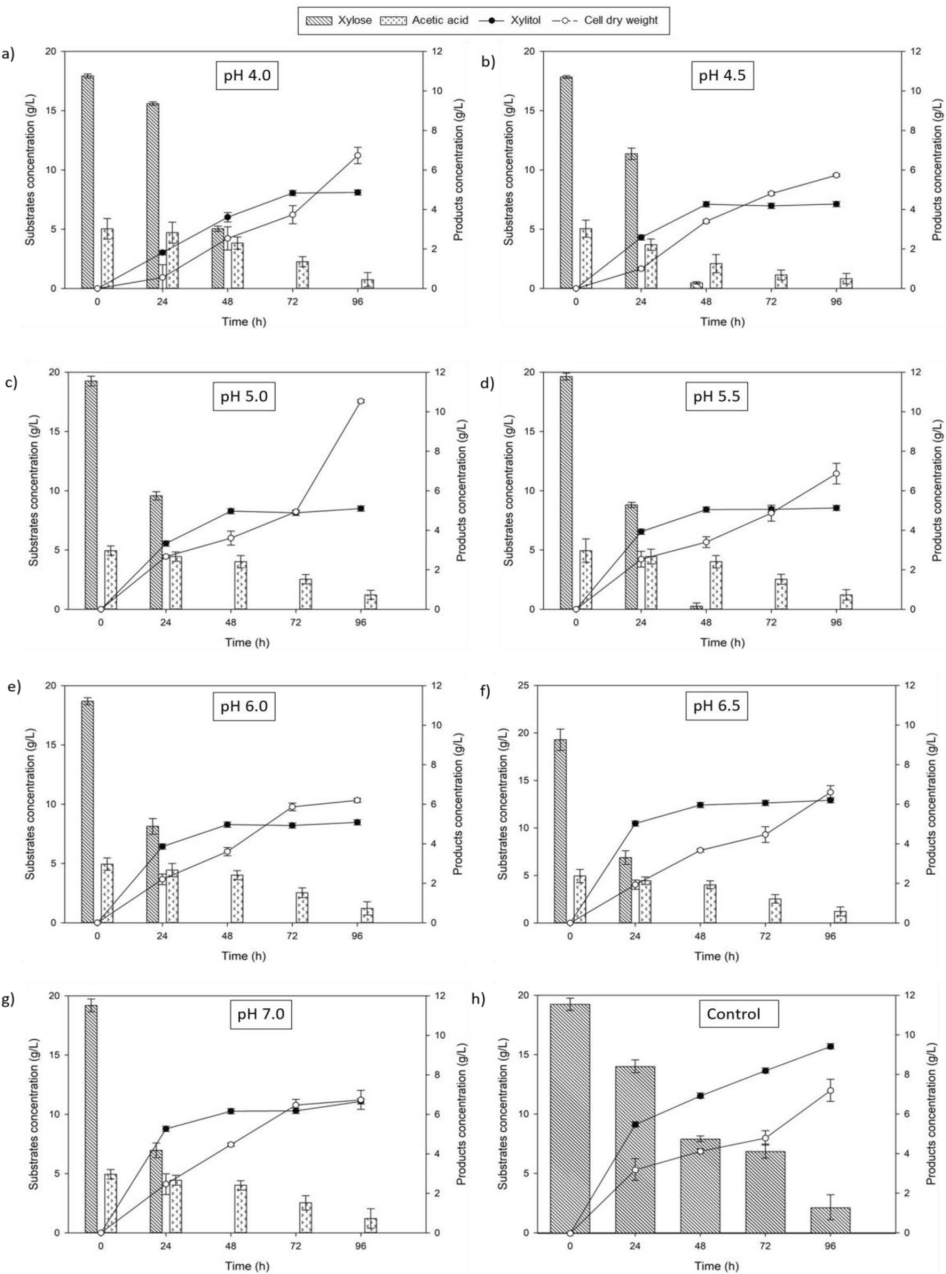
Trends of xylose bioreduction to xylitol and microbial growth of PP synthetic media by *C. tropicalis* with different initial pH levels varied from 4.0 to 7.0. The values plotted were expressed from three independent experiments expressed as mean ± standard deviation below than 1.5 g/L.

These results are consistent with the findings of prior research^30^, highlighting the significance of pH in modifying xylose consumption. As demonstrated by the study, lower pH levels are conducive to biomass proliferation, but increasing the pH from 4 to 8 led to a significant rise in the proportion of xylose consumed for xylitol formation relative to biomass growth. In this media, the carbon source is solely dependent on xylose carbon source, which needs to be utilized for the product that is needed, also for the microbes’ growth. Thus, the varied pH favors the xylose utilization either toward xylitol yield or growth. However, upon scrutinizing the trends of xylose bioreduction to xylitol and microbial growth in Fig. 2, it becomes evident that higher pH levels accelerate the utilization of xylose, shortening the time required from 48 to 24 h, especially starting at a pH of 6. This underscores the role of pH adjustments in fostering an environment conducive to expedited substrate consumption. Intriguingly, the xylitol concentration attains stability at this juncture due to the complete exhaustion of xylose. In contrast, the trajectory of biomass concentration maintains a continuous ascent, implying ongoing microbial expansion even after xylose depletion. This persistence can be attributed to the presence of nitrogen sources within the fermentation process.

The pH factor plays a critical role in creating an optimal environment that favors the production of xylitol over other byproducts. It exerts a significant influence on key determinants such as xylose transport, enzyme activity, and cell growth, all of which have a profound impact on xylitol yield and productivity^31,32^. In addition to the pH parameter, the substrates presence in the synthetic media in this study also played a crucial role, particularly concerning the presence of acetic acid as an inhibitory agent, thereby accentuating the indispensability of pH optimization in the context of xylitol production.

**Figure 3:**
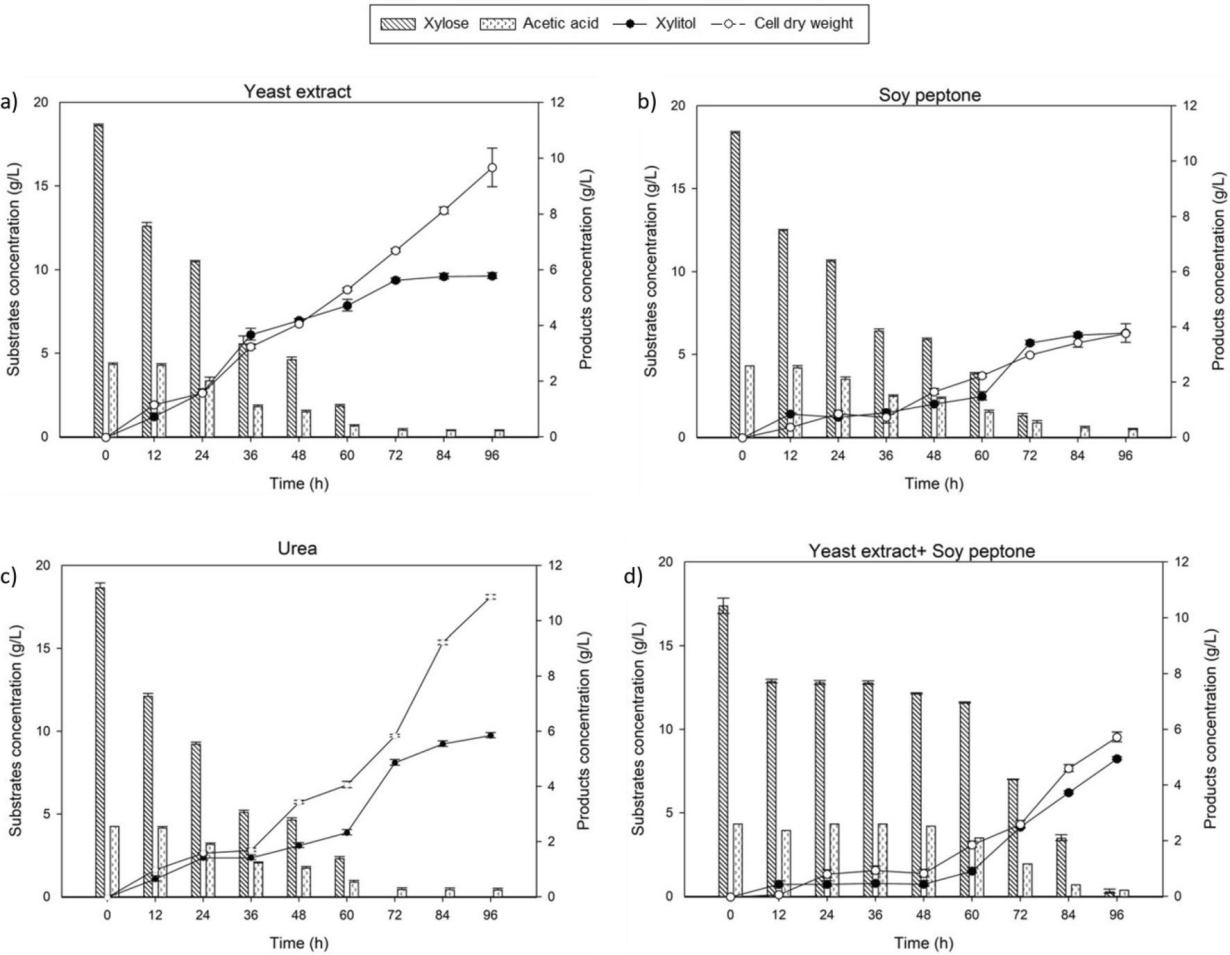
Trends of xylose bioreduction to xylitol and microbial growth of PP hydrolysate by *C. tropicalis* with different nitrogen source. The values plotted were expressed from three independent experiments expressed as mean ± standard deviation below than 1.5 g/L.

However, it’s important to note a striking difference in the control group, where acetic acid was absent. Here, xylitol concentration improved by 42%. This highlights the inhibitory role of acetic acid, even when pH is neutralized. Figure 3 clearly illustrates the contrasting trends between media with and without acetic acid. Acetic acid accelerates xylose utilization but results in lower xylitol concentration. Conversely, without acetic acid, xylose utilization is slower, and xylitol concentration steadily rises over 96 h. This phenomenon can be attributed to acetate the dissociate formed of the acetic acid providing yeast with energy through the Tricarboxylic Acid (TCA) cycle^33^, despite inhibiting xylitol production. This dual role of acetic acid presents an intriguing avenue for optimizing xylitol production. It’s essential to elucidate that the result revealed a critical relationship between pH levels and the dissociation of acetic acid. As pH increases, a higher proportion of acetic acid dissociates into acetate ions, which are readily channeled into the TCA cycle, thereby enhancing the energy supply for yeast cells. As a result, xylose utilization speeds up for xylitol production as pH levels increase.

In conclusion, optimizing pH levels is crucial for maximizing xylitol production by creating favorable conditions for enzyme activity, xylose transport, and robust cell growth in the presence of hydrolysate with acetic acid inhibitors. The results presented in this study contribute to a deeper understanding of the role of pH in xylitol production and provide valuable insights for developing strategies to optimize xylitol yield and productivity in hydrolysate with high inhibitors present. These findings align with previous research, and they offer practical implications for improving the efficiency of xylitol production processes.

### Performance of xylitol fermentation of varied nitrogen source in pineapple peel hydrolysate

Nitrogen sources play a vital role in fermentation, supporting microbial growth and metabolism as essential nutrients for various cellular processes^34,35^. However, the high cost of yeast extract, despite its superior performance as a nitrogen source, often restricts its use in large-scale fermentations. Consequently, this study was driven by the dual objectives of seeking a cost-effective alternative to yeast extract that could match its performance and exploring potential synergies among well-established nitrogen sources. This approach was not only guided by previous research, which had highlighted promising nitrogen substitutes but also aimed to investigate potential synergistic effects between these established sources.

Table 3 presents the performance of different nitrogen sources supplemented with PP hydrolysate, offering insights into their influence on xylitol production. The choice of nitrogen source significantly impacts xylose utilization, as depicted in Fig. 3. Notably, yeast extract and urea exhibited similar trends, fully utilizing xylose by 72 h and resulting in approximately ~ 6 g/L and ~ 10 g/L of xylitol concentration and dry cell weight, respectively. In contrast, soy peptone showed slower xylose utilization, reaching around 4 g/L of xylitol concentration by 84 h.

**Table 3:** Result of xylose bioreduction to xylitol and microbial growth of synthetic media by *C. tropicalis* at different pH values.

Nitrogen source	Time (h)	Xylitol concentration (g/L)	Xylose consumption (g/L)	Dry cell weight (g/L)	Xylitol yield, Y_P/S_ (g/g)	Biomass yield, Y_X/S_ (g/g)
YE	96	5.91	18.68	10.22	0.32	0.55
SP	96	3.80	18.32	3.99	0.21	0.22
Urea	96	5.76	18.49	10.92	0.31	0.59
YE + SP	96	4.98	17.21	5.78	0.29	0.34

*YE* yeast extract, *SP* Soy peptone.

The results highlight yeast extract as the dominant nitrogen source, yielding 0.32 g/g of xylitol, followed by urea (0.31 g/L), the combination of yeast extract and soy peptone (0.29 g/L), and soy peptone itself (0.21 g/L). Urea emerges as an excellent substitute for yeast extract, offering similar xylose utilization criteria with only a marginal difference in xylitol yield (0.01 g/g). This finding aligns with prior studies^36,37^ that have also demonstrated positive results using urea as a nitrogen source. The straightforward nature of urea as a nitrogen source, characterized by its composition of merely two amine groups and one carbonyl group, positions it as an attractive choice for supporting microbial growth and xylitol production. This simplicity implies that urea may not necessitate the additional complex compounds present in more intricate nitrogen sources like yeast extract. For example Ko et al.^37^ reported a 30% increase in xylitol production when substituting yeast extract with urea.

However, a contrasting finding emerged in the study by Kim and Oh^38^, where a 40% decrease in xylitol concentration compared to yeast extract was observed when using chemically defined xylose as the carbon source. This disparity between studies might be attributed to the complexity of nutrient, as when Kim and Oh^38^ added vitamins to the semi-defined medium with urea, it led to a 7% increase compared to the use of yeast extract. Therefore, it can be inferred that urea’s simplicity is particularly advantageous when working with complex hydrolysates derived from biomass pre-treatments, which contain a multitude of constituents.

As mentioned earlier, soy peptone exhibits the lowest xylitol yield with the slowest rate of xylose utilization. The rate of the xylose utilization can be related to the microbes preferability towards the nitrogen source supplied. The complexity of the nitrogen source can influence the efficiency of the transportation of the nutrients uptake of microbes^39–41^. Comparing to the urea, yeast extract and soy peptone are both organic nitrogen source which a complex nitrogen source that consists of many complicated components and nutrients. However, in this context the yeast is more prefer the yeast extract compared to the soy peptone. The reason can be due to the presence of the more complex substances like bioactive peptides in the soybeans^42^. This bioactive peptides possess specific biological activities which in this context has made it less preferable nitrogen source.

However, when the soy peptone was combined yeast extract the xylitol yield seems improving however, the xylose uptake by the microbes become more slower as even at the end of the fermentation period the xylose was not fully utilised compared when soy peptone as carbon source. As the combination of the nitrogen source make it more complex nitrogen source thus make it more slower. From this study it highlights the importance of selecting a proper and cost-effective nitrogen source, where soy peptone results in a yield of 0.21 g/g, whereas urea increases the yield to 0.31 g/g. These findings underscore the significance of optimizing nitrogen source selection to enhance fermentation performance and cost efficiency.

## Methods

### Raw material

In this study, the pineapple peel (PP) utilized is of the MD2 pineapple variant. This PP was collected from the “Kulim Pineapple Farm”, a pineapple plantation enterprise situated in Johor, Malaysia. The PP was acquired during the processing of pineapple flesh for external companies. Initially, the fresh PP was cut into small pieces, averaging 5 cm × 5 cm in size. These pieces were then left under direct sunlight for 5 days^15^. Next, the biomass was finely ground to achieve a particle size of 2 mm using a cutting mill (Model: Pulverisette 19, Fritsch, Germany). Following this, the sample was carefully dried in an oven at 60 °C for 18 h to remove humidity and maintain the moisture content’s biomass ~ 10%^43^. The prepared sample was subsequently placed in an airtight container and stored at room temperature for future applications.

### Biomass compositional analysis

**Figure 4:**
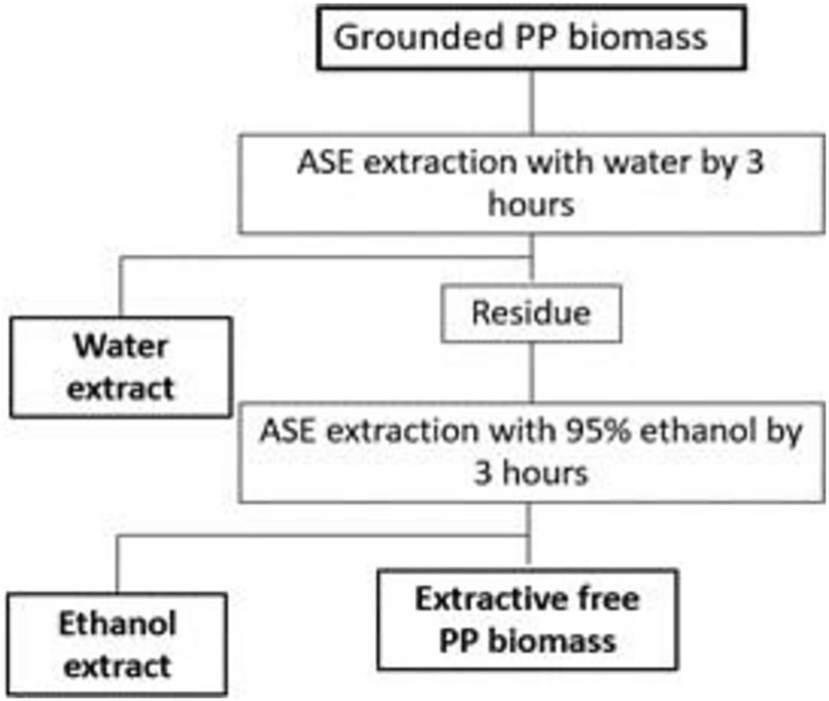
Accelerated Solvent Extraction (ASE) extraction procedures of PP for water extract, ethanol extract and extractive free biomass samples.

National Renewable Energy Laboratory (NREL), Laboratory Analytical Procedure (LAP) was used to analyse the elements of PP. The primary focus of the components is on structural carbohydrates, total extractives, and lignin. In this technique, the sample’s extractives were first eliminated entirely to prevent interference. This was accomplished through 3 h of water extraction followed by 3 h of 95% ethanol extraction using the Accelerated Solvent Extraction ASE 350 (ASE-Dionex, Sunnyvale, CA, USA)^15^. As a result, the processes produced three main samples: water extraction, ethanol extraction, and extractive free PP biomass as shown in Fig. 4. Water-extract and extract-free PP biomass samples were subjected to acid hydrolysis. High-performance liquid chromatography (HPLC) was used to assess the sugar polysaccharide from the water extract, hydrolysate of the water extract, and extract-free PP biomass. The total sugar polysaccharide from these three samples were quantified as cellulose and hemicellulose. Then, the extractive-free PP biomass hydrolysate was filtered, dried, and recorded as acid-insoluble lignin.

### One factor at a time (OFAT) approach for hydrolysis run

For the hydrolysis, nitric acid was used. Hydrolysis was performed at a 10:1 (mL: g) liquid to solid ratio by suspending 10 mL of nitric acid in 1 g of the dried biomass with moisture content less than 10%. This hydrolysis took place in a 50 mL screw capped Erlenmeyer flask. The hydrolyzed liquid was stored in a Duran bottle for later analysis using HPLC to assess various sugars and inhibitors in g/L.

OFAT method were used to determine the optimum range of the crucial hydrolysis parameters which are concentration of acid, temperature and residence time. The parameters were chosen based on the range of previous research^15^. The ideal range for those parameters are 1–7% for acid concentration, 105–130 °C for temperature and 5– 40 min for residence time^15^. OFAT hydrolysis commenced with three stages of hydrolysis. In the first stage, the hydrolysis took place by varying the acid concentrations while maintaining the temperature and residence time at 130 °C for 20 min as control. In the next stage, the best acid concentration from stage 1 was used for this hydrolysis while varying the temperature for 20 min. For the last stage, using the best acid concentration and temperature from stage 1 and stage 2, this hydrolysis was run with various residence times. The best acid concentration and the best temperature were chosen based on its ability to produce the highest sugar concentration. The overview OFAT parameter varied can be seen in Table 4.

**Table 4:** Overview OFAT hydrolysis run stages.

Stage	Nitric acid concentration (%)	Temperature (°C)	Residence time (min)
1	1–7	130	20
2	Determined from stage 1	105–130	20
3	Determined from stage 1	Determined from stage 2	10–60

### Microorganism and inoculum preparations

*C. tropicalis* FTI 20037 used in this experiment were inoculated by taking a loopful cell into 50 mL cultivation media containing (20 g/L xylose, 20 g/L peptone, 10 g/L yeast extract, 2 g/L (NH_4_)_2_SO_4_ and 0.1 g/L CaCl_2_⋅2H_2_O. The cultivation was kept in incubator at 30 °C and 200 rpm for 24 h.

### Fermentability test

In the fermentability test, two sets of batch fermentation were conducted: one pH adjustment on synthetic media of high acetic acid concentration that was designed to closely resemble the composition of the PP hydrolysate, and the other nitrogen source selection using the actual PP hydrolysate. In the pH adjustment batch fermentation experiment. The synthetic media was carefully formulated to include the main sugar, xylose, and the main inhibitor, acetic acid, obtained during the pre-treatment process, at concentrations of approximately 20 g/L and 5 g/L, respectively. The initial pH of the synthetic media was adjusted in the range of 4.0 to 7.0 with 5% (v/v) nitric acid and sodium hydroxide powder.

Once the optimal pH was determined, the fermentability test was performed using the actual PP hydrolysate. The PP hydrolysate used in this test did not undergo any prior detoxification process to retain all the inhibitors present, allowing for a better assessment of its efficiency in xylitol production as a preliminary result of the PP hydrolysate’s performance as a carbon source. After adjusting the pH of the PP hydrolysate, the reaction was left for overnight to complete all the reaction, then remove all the precipitate by centrifuged 10,000×*g* for 15 min. Subsequently, several nitrogen sources were supplemented to the media: yeast extract (control), urea, soy peptone, and a 1:1 ratio combination of yeast extract and soy peptone.

Each experiment for both sets fermentability test were conducted in 125-mL Erlenmeyer flasks with 50 mL of the working solution and inoculated at a 10% (v/v) ratio. The prepared hydrolysate was supplemented with a total of 10 g/L nitrogen source, including 2.0 g/L (NH_4_)_2_SO_4_, and 0.1 g/L CaCl_2_⋅2H_2_O.

The combination of synthetic media tests with varied pH levels and subsequent fermentability tests using the actual hydrolysate at different nitrogen sources aimed to provide valuable insights into the performance of *C. tropicalis* and optimize xylitol production from the PP waste stream. This research not only explored the influence of pH on fermentation but also investigated cost-effective alternatives for nitrogen sources in the process. The experimental setup was designed to contribute to the advancement of xylitol production from PP by improving process efficiency and cost-effectiveness.

### Analytical method

Using High Performance Liquid Chromatography, HPLC (UltiMate 3000 LC system, Dionex, Sunnyvale, CA) with a refractive index (RI) detector, the concentrations of sugar monomers and acetic acid resulting from hydrolysis were measured (RefractoMax 520, ERC, Germany). The sample was passed through a Rezex ROA-Organic acid column (300 mm 7.8 mm; Phenomenex, USA) and a guard column (50 mm 7.8 mm; Phenomenex, USA) at a temperature of 60 °C. The mobile phase, which consisted of 5 mM sulfuric acid, was eluted isocratically at 0.6 mL/min. Using HPLC, the concentrations of furfural and HMF were likewise quantified, however samples were conducted under different column and mobile phase conditions. The sample was processed on a Gemini C-18 column (Phenomenex, USA). The temperature of the column was controlled at 40 °C. The mobile phase was 20mM sulphuric acid:acetonitrile (1:10) at a flow rate of 0.8 mL/min.

## Conclusion

In conclusion, this study underscores the significant potential of PP hydrolysate as a viable carbon source for xylitol production. Optimal hydrolysis conditions, characterized by lower acid concentrations, a temperature of 105 °C, and a 20-min residence time, were identified as paramount for maximizing xylose yield from hemicellulose degradation. Importantly, fermentability tests demonstrated that the hydrolysate’s performance is on par with that of controlled media containing acetic acid. This suggests that the natural antioxidants present in PP do not hinder fermentation, with inhibitory effects primarily attributed to acetic acid. Notably, higher pH levels were found to mitigate the impact of acetic acid during fermentation, resulting in enhanced xylitol production. Furthermore, the utilization of urea as a nitrogen source emerged as a cost-effective alternative to yeast extract, yielding an impressive overall xylitol yield of 0.31 g/g. These insights offer valuable guidance for optimizing fermentation processes, enhancing efficiency, and reducing costs. Future studies could delve deeper into the variation of pH adjustment using an adjuster that could make insoluble salt rather than soluble salt, as in this study, and its impact on xylitol production. Nevertheless, it is essential to acknowledge that the prominent of detoxification process takes place, as the presence of acetic acid, even with the best pH adjustment, causes approximately a 30% reduction in xylitol performance. Investigating novel detoxification techniques and their impact on xylitol production could enhance the overall efficiency of the process. Beyond these technical benefits, this study also addresses environmental concerns and economic opportunities. It provides a sustainable solution for managing PP waste, which is a pertinent issue in Malaysia, and opens doors for the pineapple industry to generate substantial income by valorizing its residual resources. In essence, this research makes a substantial contribution to the field by showcasing the untapped potential of PP biomass for xylitol production. By doing so, it not only delivers economic benefits but also aligns with environmental sustainability goals and helps resolve waste management challenges faced by the pineapple industry. To fully realize this potential, further investigations and assessments of the scalability of the fermentation process using PP hydrolysate for industrial applications are warranted.

### Ethics statement

In accordance with ethical principles and legal requirements, this research involving pineapple peel (PP) utilized plant materials responsibly and in compliance with institutional, national, and international guidelines.

## Data Availability

All data generated or analysed during this study are included in this published article.
